# Sex-Specific Health and Economic Benefits in Older Women at Risk of Atrial Fibrillation: A Proof-of-Concept Evaluation of an AI-Enabled Strategy for Early Thromboembolic Risk Detection

**DOI:** 10.3390/jcm15082861

**Published:** 2026-04-09

**Authors:** Anna Panisello-Tafalla, Josep L. Clua-Espuny, Eulalia Muria-Subirats, Josep Clua-Queralt, Jorgina Lucas-Noll, Teresa Forcadell-Arenas, Silvia Reverte-Villarroya

**Affiliations:** 1 Equip d’Atenció Primària Tortosa Est, Gerència d’Atenció Primària i a la Comunitat de les Terres de l’Ebre, Institut Català de la Salut, 43500 Tortosa, Spain; apanisellot.ebre.ics@gencat.cat (A.P.-T.); jcluaq@gmail.com (J.C.-Q.); 2 Fundació Institut Universitari per a la Recerca a l’Atenció Primària Jordi Gol i Gurina (IDIAPJGol), 43500 Tortosa, Spain; 3 Equip d’Atenció Primària Amposta, Gerència d’Atenció Primària i a la Comunitat de les Terres de l’Ebre, Institut Català de la Salut 43870 Amposta, Spain; emuria.ebre.ics@gencat.cat; 4 Hospital Comarcal Mora d’Ebre, Salut Terres de l’Ebre, 43740 Mora d’Ebre, Spain; jlucas.hcme.ste@gencat.cat; 5 Equip d’Atenció Primària Tortosa Oest, Gerència d’Atenció Primària i a la Comunitat de les Terres de l’Ebre, Institut Català de la Salut, 43500 Tortosa, Spain; tforcadella.ebre.ics@gencat.cat; 6 Advanced Nursing Research Group, Nursing Department, Rovira i Virgili University, Biomedicine Doctoral Programme Campus Terres de l’Ebre, Av. De Remolins, 13, 43500 Tortosa, Spain

**Keywords:** atrial fibrillation, thromboembolic risk, artificial intelligence, stroke prevention, women’s cardiovascular health, sex differences, primary care, cost-effectiveness analysis, digital health, quality-adjusted life-years (QALYs), health equity, electronic health records

## Abstract

**Background**: Women with atrial fibrillation experience a higher lifetime risk of ischemic stroke, greater stroke severity, and worse functional outcomes than men. Preventive strategies focused on AF detection may therefore miss critical opportunities for early intervention in women. **Methods**: We developed a decision-analytic Markov model using real-world primary care data from Catalonia (Spain) to evaluate an artificial intelligence (AI) enabled strategy for upstream thromboembolic risk detection. The intervention combined electronic health record–based risk prediction, targeted digital rhythm screening, and individualized anticoagulation. Lifetime clinical and economic outcomes were estimated for adults aged ≥65 years, with pre-specified sex-stratified analysis. **Results**: Compared with usual care, the AI-enabled strategy reduced ischemic stroke, major adverse cardiovascular events, and long-term disability. Absolute reductions in stroke and disability were greater in women, reflecting higher baseline thromboembolic risk. Per 1000 high-risk women, the strategy prevented more strokes and generated larger quality-adjusted life-year gains than in men. From both healthcare payer and societal perspectives, the intervention was cost-saving in women, driven by reductions in stroke-related disability and long-term care. **Conclusions**: AI-enabled upstream thromboembolic risk detection may deliver particularly important benefits for older women and represents a promising approach to reduce sex-based inequities in stroke prevention.

## 1. Introduction

Atrial fibrillation is the most common sustained cardiac arrhythmia and a major contributor to ischemic stroke, particularly in older adults. AF-related strokes are typically more severe, associated with higher disability, and generate substantial long-term healthcare and societal costs [1,2,3]. Importantly, a considerable proportion of thromboembolic events occur in individuals without a prior diagnosis of AF, suggesting that current prevention strategies may be implemented too late in the disease trajectory [4].

These challenges are especially relevant in women. Compared with men, women experience AF at older ages and have a higher lifetime risk of ischemic stroke, worse functional outcomes, and greater long-term disability, even after adjustment for comorbidities [2,3]. In addition, women are more likely to experience delayed diagnosis, less intensive rhythm monitoring, and lower rates of timely anticoagulation initiation [5,6]. These disparities reflect broader inequities in cardiovascular care, partly driven by the historical under-representation of women in clinical research and the use of risk prediction tools derived from predominantly male populations [7,8,9].

Current clinical practice relies heavily on risk stratification tools such as the CHA_2_DS_2_-VA score to guide anticoagulation decisions once AF has been diagnosed [10]. However, these tools were developed in populations with established AF and are not designed to assess thromboembolic risk in earlier, pre-clinical stages. Consequently, they may inadequately capture important determinants of risk—such as multimorbidity, frailty, inflammation, and age-related atrial remodeling—which are highly prevalent in older individuals and particularly relevant in women [11]. As a result, many individuals may remain classified as low or intermediate risk until experiencing a first, often disabling, thromboembolic event.

Emerging evidence suggests that thromboembolic risk may precede clinically manifest AF and may be driven by underlying atrial cardiomyopathy and systemic factors [12,13]. Recent clinical guidelines have also introduced the concept of “at-risk” or “pre-AF” stages, recognizing a potential window for earlier preventive intervention [14]. However, current prevention strategies remain largely focused on detecting overt arrhythmia rather than identifying upstream risk.

In this context, artificial intelligence (AI) offers new opportunities to integrate large-scale electronic health record (EHR) data and identify individuals at high thromboembolic risk before the onset of clinically apparent AF. AI-based models can incorporate multidimensional clinical information, including comorbidities and longitudinal health data, potentially improving risk stratification beyond traditional clinical scores [15,16].

The MATHIAS project (“throMboembolic risk Associated To High atrIal fibrillation riSk”) was developed to address this gap by applying machine learning techniques to routinely collected primary care data in adults aged ≥65 years at high risk of AF [15,16,17]. By integrating demographic, clinical, and multimorbidity-related variables, the model aims to identify individuals—particularly women—at increased thromboembolic risk prior to AF diagnosis.

The aim of this study is to evaluate the long-term clinical and economic impact of an AI-enabled upstream thromboembolic risk detection strategy compared with usual care, with a specific focus on sex-specific outcomes and implications for women’s cardiovascular health [18].

## 2. Materials and Methods

### 2.1. Study Design and Cohort

We conducted a model-based economic evaluation informed by a retrospective observational cohort from the SAPiC Terres de l’Ebre primary care database (Catalonia, Spain). The study combines real-world data analysis with a decision-analytic Markov model to estimate long-term clinical and economic outcomes, in accordance with CHEERS 2022 reporting standards and Nature Research reporting standards regarding transparency, relevance, and interpretability of model-based analysis. All analyses were conducted using pseudo-anonymized data, and no individual-level treatment decisions were made within the study framework, ensuring compliance with applicable ethical standards and data protection requirements. A cohort state-transition Markov model compared to an AI-enabled digital prevention pathway with usual care over a lifetime horizon.

The AI model MATHIAS was developed to predict thromboembolic events (MACE, stroke, and systemic embolism) in older adults at high AF risk using routinely collected primary care EHR data [18]. Candidate predictors included demographics, cardiovascular risk factors, comorbidities, prior events, laboratory parameters, medication use, and risk scores such as CHA_2_DS_2_-VA and Charlson index, extracted from structured EHR fields and ICD-10 codes. The model was trained and internally validated in a population-based AF cohort previously described, using a train-validation split at the patient level. Several machine learning algorithms were explored (gradient-boosted trees, random forests, penalized logistic regression), and the final MATHIAS model was selected based on discrimination AUC, calibration, and clinical interpretability [15].

### 2.2. Data Sources and Population

This study used routinely collected data from the SAPiC Terres de l’Ebre primary care database (Catalonia, Spain) (Figure S1. Terres Ebre Map), covering 178,112 inhabitants (49.6% women) managed in 11 primary care health centers. The region is characterized by advanced population aging [19] (aging index 159.5 vs. 131.3 in Catalonia and 118.4 in Spain) and lower average per capita income [20] (77.4% of the Catalan mean). Eligible participants were adults aged 65–95 years without prior atrial fibrillation and with active electronic health records between 1 January 2015 and 31 December 2024. Individuals with previously documented AF were excluded. Additional exclusions included patients with incomplete key clinical data required for risk estimation, where applicable. This cohort is characterized by multimorbidity and high-predicted risk of AF and related complications, reflecting patients typically managed in primary care in European health systems, and provides a real-world setting with a high cardiovascular burden and constrained resources. MACE were defined as the first occurrence of a composite cardiovascular endpoint comprising myocardial infarction, stroke, extracranial systemic embolic events (SEEs), or cardiovascular death [8].

### 2.3. Intervention

Two strategies were compared:

MATHIAS-guided strategy (intervention): estimates individual thromboembolic risk; the target population was defined as individuals within the highest quartile (Q4) of predicted AF risk. This stratification was performed using a validated risk model—comprising age, sex, hypertension, diabetes, vascular disease, heart failure, and body mass index—which demonstrated robust predictive performance (AUC 0.78; 95% CI 0.75–0.81) [21,22]. The process included a subsequent clinical evaluation and device-based photoplethysmography screening [8,23], followed by AI-driven thromboembolic risk stratification using the MATHIAS AI prototype [15,16].

Usual care (comparator): Opportunistic AF detection during routine clinical encounters and anticoagulation guided by the CHA_2_DS_2_VA score in patients with documented AF, without any AI-based pre-AF risk assessment. This approach reflects current guideline-concordant practice in many European primary care settings, where digital AF screening has not yet been implemented. Opportunistic AF detection during routine clinical encounters and anticoagulation guided by the CHA_2_DS_2_-VA.

### 2.4. Considerations of AI Use

Sex-disaggregated analysis was conducted for predicted thromboembolic risk, major adverse cardiovascular events, stroke, disability outcomes, quality-adjusted life-years (QALYs), and costs. This approach was intended to identify potential differences in model performance and downstream impact between women and men and to assess whether the AI-enabled strategy modified existing sex-based disparities in cardiovascular prevention.

The development of the MATHIAS model leveraged anonymized primary care EHR data to ensure high external validity and minimize bias. The study population comprised a real-world cohort of adults aged 65 and older, ensuring the model’s applicability to clinical practice. Comprehensive descriptions of this data source have been previously peer-reviewed and published [5,8,15,17,18,21,24]. Model input variables extended beyond traditional thromboembolic risk scores to include demographic and multimorbidity-related factors relevant to both sexes. The AI-based risk estimates generated by MATHIAS were incorporated into the economic model as a decision support input rather than an automated decision-making mechanism.

### 2.5. Decision-Analytic Model

A cohort state-transition (Markov) model with annual cycles was developed to project long-term clinical and economic consequences of implementing the AI-enabled digital pathway versus usual care in the high-risk cohort. The model included six mutually exclusive health states: (1) high risk without AF, (2) pre-AF substrate, (3) detected AF, (4) post-MACE, (5) disability and dependency, and (6) death. In each yearly cycle, individuals could develop AF, experience a MACE, transition to disability, or die; death was modeled as an absorbing state. A lifetime horizon (up to death or age 95 years) was chosen to capture the full downstream impact of stroke, disability, and survival.

Transition probabilities for AF, stroke, heart failure, chronic kidney disease, peripheral arterial disease, cognitive impairment, MACE, and all-cause mortality were derived from the SAP Terres de l’Ebre cohort (Table S1 Cohort ≥ 65-year-old Terres Ebre) and calibrated to reproduce observed event rates in the MATHIAS population. Structural assumptions included: (i) a constant relative effect of MATHIAS on thromboembolic risk identification over time, (ii) similar adherence to anticoagulation under both strategies once initiated, and (iii) no explicit modeling of anticoagulation related major bleeding beyond its contribution to observed net clinical events. Model validation involved comparing the predicted incidence of AF, stroke, and MACE against observed data in the underlying cohorts and checking that long term survival patterns were plausible for similar high-risk populations. A budget impact framework was used to estimate the short to medium term financial consequences of MATHIAS score implementation over 3–5 years, balancing additional screening and anticoagulation costs against savings from avoided AF related strokes and disability.

To illustrate how the clinical and economic value of the MATHIAS strategy is distributed across the high-atrial fibrillation risk population, a Population Impact Plot (PIP) was constructed. The PIP integrates individual thromboembolic risk with population size and downstream value. Sex-specific PIPs were generated using identical risk stratification thresholds and model structure, applying sex-specific baseline risks, costs, and utilities. This approach allows for the visualization of the trade-off between clinical severity and population reach, and highlights which subgroups contribute most to overall health gains and economic value. The high-risk population was stratified into three clinically meaningful subgroups (very-high-risk, high-risk, and moderate-to-high-risk) based on the baseline CHA_2_DS_2_-VA score and the presence of a pre-atrial fibrillation substrate, and prior thromboembolic events for each stratum, the Markov model was used to estimate the absolute number of events avoided, quality-adjusted life-years (QALYs) gained, and cost savings associated with the MATHIAS strategy compared with usual care over a lifetime horizon.

### 2.6. Outcomes, Economic Perspective, and Uncertainty Analysis

Economic outcomes included direct medical costs (screening, monitoring, anticoagulation, acute events, and long-term disability) from a healthcare payer perspective and additional informal caregiving, long-term social care, nursing-home costs, and productivity losses (for individuals <75 years) from a societal perspective. Costs and QALYs were discounted at 3% annually, in line with European health-economic guidance, and results were reported per 1000 high-risk individuals [23,24,25].

Model development and analysis were performed using IBM SPSS Statistics 19 and Python version 3, taking advantage of the Scikit-learn (SKLearn version 1.7) and libraries for its versatility, performance, and ease of programming. Probabilistic sensitivity analysis was performed using 10,000 Monte Carlo simulations, with parameter uncertainty modeled using beta distributions for probabilities and utilities, gamma distributions for costs, and log-normal distributions for relative risks. Cost-effectiveness acceptability curves were generated across willingness-to-pay thresholds, including €30,000 per QALY. Deterministic one–way sensitivity analysis varied key drivers—stroke unit costs, prevalence of pre-AF substrate, MATHIAS sensitivity/specificity, and disability rates—within plausible ranges.

To examine heterogeneity, sex-stratified models were run using sex-specific baseline characteristics, event rates, and, where available, costs and utilities, while keeping the model structure, horizon, and discounting identical.

## 3. Results

### 3.1. Cohort Characteristics and AI Model Performance

In the base-case scenario (Table 1), the cohort included 9677 individuals aged 65–95 years (≈31% of the regional population ≥65 years) at high-risk of atrial fibrillation, with women representing a slightly older subset with a high burden of multimorbidity, including frequent hypertension, diabetes, vascular disease, heart failure, chronic kidney disease, and prior cerebrovascular events, and an elevated CHA_2_DS_2_-VA score.

At baseline, women showed a higher predicted thromboembolic risk and a greater burden of functional dependency following cardiovascular events (Table 2). Implementation of the MATHIAS strategy resulted in a higher proportion of individuals identified as high-thromboembolic-risk compared with usual care, with a more pronounced effect in women. This translated into the earlier initiation of preventive interventions and a reduction in downstream adverse cardiovascular outcomes.

### 3.2. Clinical Outcomes

Compared with usual care (Table 3), the AI-enabled strategy was associated with a reduction in MACE, particularly ischemic stroke. The absolute and relative reductions were consistently greater in women than in men. Per 1000 high-risk individuals, the MATHIAS-guided strategy reduced total MACE by approximately 30–40%, AF-related ischemic stroke by 25–35%, and severe post-stroke disability by 20–30% compared with usual care. These reductions generated an estimated gain of 74 additional QALYs per 1000 individuals.

**Figure 1:**
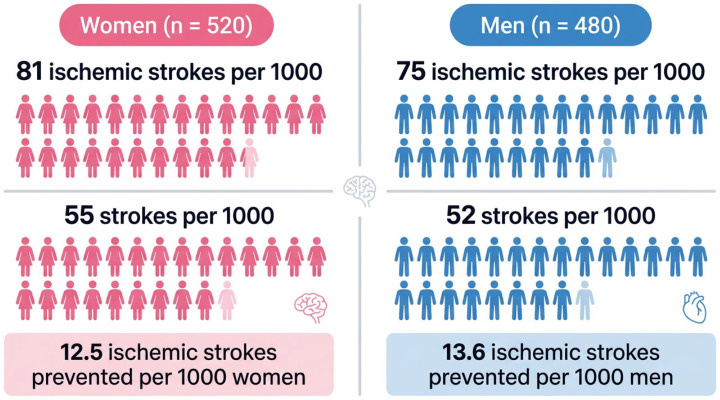
Impact of the MATHIAS-guided strategy on ischemic stroke prevention in a hypothetical cohort of 1000 high-risk individuals aged ≥65 years.

Figure 1 presents an exploratory Population Impact Plot (PIP) illustrating the projected impact of the MATHIAS-guided strategy on ischemic stroke prevention, stratified by sex, in a hypothetical cohort of 1000 high-risk individuals aged ≥65 years.

Based on the modeled cohort composition (48% men and 52% women), and assuming a similar relative effectiveness of the MATHIAS strategy in both sexes, the intervention was associated with a substantial reduction in the absolute number of ischemic strokes compared with usual care. Under usual care, approximately 78 ischemic strokes per 1000 individuals were expected over the lifetime horizon, compared with 52 strokes per 1000 with the MATHIAS strategy, corresponding to 26 strokes prevented overall.

When disaggregated by sex, this translated into approximately 12.5 ischemic strokes prevented per 1000 men and 13.6 ischemic strokes prevented per 1000 women. The slightly higher absolute number of prevented events among women reflects their greater representation in the target population rather than a difference in relative treatment effect. Reductions in cardiovascular mortality and dementia-related outcomes followed a similar pattern, with more favorable absolute gains observed among women.

### 3.3. Costs and Cost-Effectiveness

From the healthcare payer perspective (Table 4), the AI-enabled strategy was associated with higher costs related to screening and preventive treatment, partially offset by reductions in long-term stroke-related care.

Women larger savings, €1.4 million per 1000 women, related to long-term disability and post-stroke care, consistent with their higher baseline risk of severe stroke outcomes. Men accrued greater savings (€1.0 million per 1000 men) from avoided MACE hospitalizations and reduced cardiovascular mortality. In both sexes, MATHIAS remained cost saving from the healthcare payer perspective. Total direct medical cost would decrease by 1.28 million per 1000 high-risk individuals and make the pathway a dominant strategy.

From the societal perspective (Table 5), savings were amplified in women due to reductions in long-term care, nursing home admissions, and informal caregiving.

Aggregate societal savings of €1.1–€1.3 million per 1000 high-risk individuals would reinforce the dominance of the AI-enabled pathway, particularly in women, in whom reductions in long-term dependency and care-giving needs were most pronounced. In men, savings were distributed across reduced caregiving needs and avoided premature mortality, with consistently favorable economic outcomes. Across both sexes, the inclusion of societal costs strengthened the probability that MATHIAS is not only cost-effective but also cost-saving.

In probabilistic sensitivity analysis, the AI-enabled digital pathway had an estimated 0.88 probability of cost-effectiveness at a threshold of €30,000 per QALY and a 0.73 probability of being cost-saving from the payer perspective (Table 6).

Sex-stratified analysis indicated larger absolute reductions in stroke and disability—and consequently higher QALY gains and cost savings—in women, who have a higher baseline thromboembolic and disability risk, with estimated lifetime savings of around €1.4 million per 1000 women. Men experienced greater absolute mortality reductions and remained in the cost-saving, dominant region, with slightly lower QALY gains and savings of roughly €1.0 million per 1000 men.

Probabilistic sensitivity analysis confirmed the robustness of the results, with a high probability of cost-effectiveness across a wide range of parameter uncertainty. Cost-effectiveness acceptability curves showed consistently higher probabilities of cost-effectiveness for women compared with men at all willingness-to-pay thresholds considered. Deterministic sensitivity analysis identified baseline thromboembolic risk and stroke-related costs as the main drivers of cost-effectiveness, without altering the direction of sex-specific results.

### 3.4. Population Impact Plot and Distribution of Benefits

**Figure 2:**
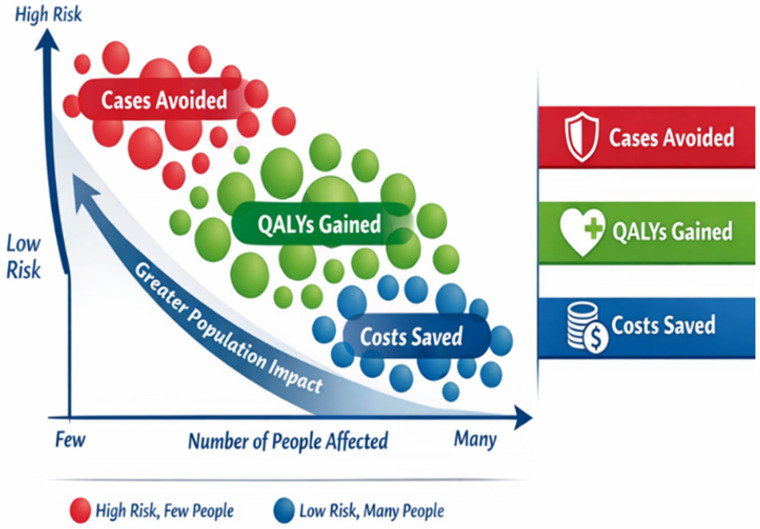
MATHIAS Population Impact Plot.

The Population Impact Plot (PIP) illustrated how clinical and economic benefits were not uniformly distributed across risk strata (Figure 2). The value generated by the MATHIAS strategy was not uniformly distributed across risk strata.

The plot demonstrates that the highest absolute impact—measured by strokes and severe disabilities avoided per capita—would be achieved in the highest-risk subgroup (pre-AF substrate and/or CHA_2_DS_2_-VA ≥ 4 or prior stroke). Intermediate-risk groups contributed the largest share of total QALYs gained and cost savings due to their greater population size. Sex-specific PIPs revealed greater absolute impact in women, driven by their higher baseline thromboembolic risk and dependency. Men, however, derived proportionally greater mortality benefits.

## 4. Discussion

Our findings are broadly consistent with prior economic evaluations of atrial fibrillation screening, which have reported stroke reductions ranging between 20% and 35% depending on population risk and screening intensity [26,27,28]. The magnitude of stroke reduction observed in our model (≈33%) falls within this range, supporting the plausibility of the projected benefits. However, unlike conventional screening approaches that rely on arrhythmia detection, the MATHIAS strategy targets upstream thromboembolic risk, which may explain the larger impact on disability outcomes observed in our study.

Compared with machine learning-based AF prediction models evaluated in previous studies [25,26], our approach extends the scope from AF detection to thromboembolic risk stratification. This distinction is clinically relevant, as emerging evidence suggests that thromboembolic risk may precede AF diagnosis and may not be fully captured by AF-centered strategies [29]. In this context, our findings align with the hypothesis of an underlying atrial cardiomyopathy contributing to stroke risk independently of overt AF.

Importantly, while prior studies have generally reported similar relative benefits across sexes, our results highlight larger absolute gains in women. This is consistent with epidemiological data showing higher stroke severity and disability burden in women [4,5,6], but it contrasts with traditional screening paradigms that do not explicitly address sex-specific risk trajectories. These findings suggest that AI-enabled risk stratification may help reduce, rather than reinforce, existing sex disparities, although this hypothesis requires prospective validation.

Nevertheless, our results should be interpreted with caution. Some studies have raised concerns that AI-based tools may inadvertently amplify existing biases if not properly validated across subgroups [30]. In our model, equal relative effectiveness was assumed for men and women, which may not reflect real-world differences in treatment response or healthcare access. Future studies should explicitly evaluate sex-specific performance and fairness metrics.

This study demonstrates that an AI-enabled upstream thromboembolic risk detection strategy may provide benefits for older women, a population historically underserved by AF-centered prevention approaches. Larger absolute reductions in stroke and disability in women highlight the importance of early risk identification and prevention. By preventing disabling strokes, the intervention not only improves health outcomes but also reduces long-term dependency and care-giving burden—outcomes of particular relevance to women’s health and social equity.

Using a risk prediction algorithm to selectively screen the highest risk individuals may be more efficient and cost effective than conducting traditional systematic or opportunistic screening [31]. The number needed to screen to detect AF is markedly lower than in unselected populations [8,21]. Emerging evidence suggests that a prothrombotic atrial myopathy may precede AF and independently drive thromboembolic risk; and individuals with a high predicted AF risk can experience MACE similar incidence to rates observed in populations with established AF [18,29]. In model projections, this strategy reduced MACE, ischemic stroke, and severe post-stroke disability and generated meaningful QALY gains, while being cost saving in most scenarios.

The sex-stratified analysis showed broadly similar relative benefits for women and men but larger absolute QALY gains and cost savings in women, driven by a higher baseline thromboembolic and disability risk. These patterns suggest that prioritizing high-risk older women for AI-guided assessment and digital screening may be both efficient and equity-enhancing, potentially helping to mitigate long-standing sex disparities in stroke prevention [32]. At the same time, they underscore the need to carefully monitor algorithmic performance across demographic subgroups and to ensure that AI-enabled pathways do not exacerbate existing inequities in access to digital health tools or anticoagulation. Future work should therefore include fairness-oriented evaluations, examining calibration, error rates, and realized benefits across sex, age, socioeconomic status, and other relevant axes of vulnerability, such as a limited digital literacy or access [29,30,33].

### 4.1. Strengths and Limitations of the AI-Enabled Modeling Approach

Importantly, this study should be interpreted as a proof-of-concept evaluation rather than evidence of clinical effectiveness. The primary contribution of this work lies in demonstrating the plausibility and potential value of an AI-enabled approach that targets thromboembolic risk upstream of clinically overt atrial fibrillation—a risk domain not explicitly addressed by current guideline-recommended tools such as CHA_2_DS_2_-VA. By incorporating a broader set of routinely available EHR variables, MATHIAS captures dimensions of risk related to multimorbidity, frailty, and systemic vulnerability that may be particularly relevant in older primary care populations. The simulated reductions in stroke, disability, and societal costs should therefore be viewed as indicative estimates that support the prioritization of external and prospective validation, rather than as claims of immediate translational impact. The sex-stratified PIP should be interpreted as an exploratory scenario analysis.

Several limitations of this study should be carefully considered. First, although the MATHIAS model demonstrated adequate internal validation, it has not yet undergone external validation in independent populations. This represents a key limitation, as model performance, calibration, and generalizability may differ across healthcare settings, populations, and data structures. This study did not directly assess usability, acceptability, or workflow integration in clinical practice, nor did it evaluate patient-reported outcomes or behavioral responses to AI-generated risk information. While our current findings provide valuable insights, the lack of CKD staging represents a methodological limitation. Thromboembolic risk and cardiovascular outcomes are closely linked to the severity of renal impairment, suggesting that a more detailed stratification could further refine model precision. This complexity has been addressed in our recent work [34] where machine learning algorithms were employed to specifically evaluate the interplay between CKD risk levels and MACE in patients with atrial fibrillation. These implementation and human-factors dimensions are critical for realizing the potential of AI-enabled digital pathways in routine care.

Second, the decision-analytic framework necessarily relies on structural assumptions that simplify complex real-world processes. In particular, the model assumes a constant effect of the intervention over time, which may not fully reflect clinical reality. In practice, the effectiveness of an AI-enabled strategy may vary due to factors such as patient adherence, clinician uptake, treatment initiation delays, and healthcare system characteristics. These dynamics could influence both clinical outcomes and cost-effectiveness estimates.

Third, additional simplifications include the representation of care pathways, the absence of explicit modeling of certain adverse events (e.g., anticoagulation-related bleeding beyond net clinical outcomes), and the reliance on aggregated transition probabilities. While these assumptions are consistent with standard modeling approaches, they introduce uncertainty and may affect the precision of the estimates.

Finally, although the model was calibrated using real-world data, the projections remain hypothetical and should be interpreted as exploratory and hypothesis-generating.

### 4.2. Generalizability and Validation of Roadmap

The present study represents an early, hypothesis-generating step in the emerging frameworks for digital health evaluation, focusing on algorithm development, internal validation, and system-level impact modeling, rather than on clinical effectiveness or implementation outcomes. A structured validation of roadmap is, therefore, required before any consideration of clinical deployment [26]. First, external validation in independent primary care cohorts is essential to assess discrimination, calibration, and fairness across different populations, EHR systems, and care contexts. Second, prospective implementation studies should evaluate how MATHIAS performs when embedded within real-world EHR workflows, including its impact on AF detection rates, anticoagulation initiation, and clinician decision-making. Third, evaluation of real-world clinical outcomes and workflow integration trials will be necessary to determine whether the projected reductions in stroke, disability, and costs translate into measurable improvements in patient outcomes and health-system efficiency. In parallel, ongoing model updating, performance drift, and explicit evaluation of algorithmic fairness will be required to ensure safe and equitable use. Within this framework, the current results should be viewed as providing a quantitative rationale for prioritizing further validation of AI-enabled upstream thromboembolic risk stratification, rather than as evidence supporting immediate clinical adoption.

### 4.3. Implications for Women’s Health Policy and Primary Care

To our knowledge, few studies have evaluated the clinical and economic impact of strategies targeting thromboembolic risk upstream of atrial fibrillation diagnosis in individuals at high risk of AF. Most existing evidence has focused on AF screening or early detection strategies—either opportunistic or systematic—which aim to identify previously undiagnosed AF and initiate anticoagulation based on established risk scores. These approaches have demonstrated variable effectiveness and cost-effectiveness, largely depending on population risk profiles and screening intensity, but remain centered on the detection of overt arrhythmia [27,28,35,36].

Although several artificial intelligence models have been developed to predict incident atrial fibrillation or major cardiovascular events, most focus on arrhythmia detection or short-term risk prediction [25,35,36] and have not focused on upstream thromboembolic risk as a primary target for intervention. In contrast, the MATHIAS approach targets thromboembolic risk in individuals without diagnosed AF and integrates these predictions into a long-term economic modeling framework. To our knowledge, no previous studies have combined AI-based upstream risk stratification with sex-specific cost-effectiveness analysis, limiting direct comparability but highlighting the innovative nature of this approach.

The findings of this study have relevant implications for women’s health policy and for the organization of cardiovascular prevention in primary care. By demonstrating that an AI-enabled upstream thromboembolic risk assessment strategy may yield greater clinical and economic benefits in women than in men, the results highlight the need to move beyond sex-neutral prevention frameworks that may inadvertently perpetuate inequities. Integrating AI-based decision support tools such as MATHIAS into routine primary care workflows could facilitate earlier identification of women at high thromboembolic risk, particularly older women with multimorbidity who are frequently under-recognized by traditional risk scores.

From a policy perspective, strategies that prioritize early, sex-sensitive risk assessment have the potential to reduce stroke-related disability, long-term dependency, and informal caregiving burden among women, aligning with broader public health goals of equity, sustainability, and value-based care. Importantly, the use of AI in this context should be accompanied by robust governance frameworks that ensure transparency, clinician oversight, and continuous monitoring of sex-specific performance, reinforcing its role as a tool to support—not replace—clinical judgment in cardiovascular prevention.

## 5. Conclusions

AI-enabled upstream thromboembolic risk detection may substantially reduce stroke-related morbidity and generate economic savings, with particularly important benefits for older women. These findings support the idea that sex informed AI prevention strategies could represent a powerful tool to reduce cardiovascular inequities and improve healthy aging in women.

Future research should focus on external validation of the model in independent populations, prospective clinical studies to assess real-world effectiveness, and the evaluation of implementation strategies within routine primary care settings.

## Figures and Tables

**Table 1:** Demographic and clinical characteristics of the simulated cohort at model entry, stratified by sex.

Variables	MEN	(%)	WOMEN	(%)	*p*	ALL (%)
All (%)	4231	43.72%	5446	56.27%		9677
Age average	84.6 ± 6.9		84.7 ± 6.7		0.474	84.66 ± 6.76
65–74 years	131	3.1%	97	1.78%		228 (2.35%)
≥75 years	4100	(96.90%)	5349	98.21%		9449 (97.64%)
CHA_2_DS_2_-VA	4.10 ± 0.97		3.84 ± 0.88		<0.001	3.96 ± 0.9
New AF	863	20.4%	851	15.62%	<0.001	1714 (17.7%)
Heart failure	1236	29.2%	1415	26.0%	<0.001	2651 (27.4%)
Hypertension arterial	3708	87.6%	4916	90.3%	<0.001	8624 (89.1%)
Diabetes mellitus	2261	53.4%	2414	44.3%	<0.001	4675 (48.3%)
Stroke/TIA/Systemic embolism	458	10.8%	496	9.1%	0.005	954 (9.9%)
Peripheral vascular disease	1025	24.2%	619	11.4%	<0.001	1644 (17.0%)
Ischemic heart disease	1100	26.0%	714	13.1%	<0.001	1814 (18.7%)
BMI ^1^ (kg/m^2^)	30.2 ± 5.0		31.23 ± 5.9		<0.001	30.78 ± 5.5
Charlson index	2.54 ± 1.5		1.96 ± 1.3		<0.001	2.21 ± 1.4
Dementia/cognitive impairment	616	14.6%	1173	21.5%	<0.001	1789 (18.5%)
Pfeiffer score	2.87 ± 3.0		3.93 ± 3.1		<0.001	3.54 ± 3.1
Chronic Kidney Disease	1469	34.7%	1881	34.5%	0.863	3350 (34.6%)
Glomerular filtration rate (mL/min/1.73 m^2^)	60.55 ± 19.6		60.28 ± 19.6		0.623	60.4 ± 19.6
OSAHS ^2^	274	6.5%	160	2.9%	<0.001	434 (4.5%)
Dyslipidaemia	2079	49.1%	3104	57.0%	<0.001	5183 (53.6%)
Statins	1436	33.9%	1664	30.6%	<0.001	3100 (32.0%)
Antiplatelet therapy	1098	26.0%	1079	19.8%	<0.001	2177 (22.5%)
Anticoagulation	792	18.7%	772	14.2%	<0.001	1564 (16.2%)
VKAs ^3^	330	7.8%	318	5.8%	<0.001	648 (6.7%)
NOACs ^4^	463	10.9%	457	8.4%	<0.001	920 (9.5%)
Hospital visits	0.56 ± 1.51		0.40 ± 1.21		<0.001	0.48 ± 1.43
Active medications	7.07 ± 4.7		7.32 ± 4.6		0.009	7.38 ± 4.9
Death all-causes	2456	58.0%	3543	65.1%	<0.001	5999 (62.0%)

^1.^ BMI: Body Mass index; ^2^ OSAHS: Obstructive Sleep Apnea-Hypopnea Syndrome; ^3^ VKAs: vitamin K antagonists; ^4^ NOACs: Non-vitamin K Antagonist Oral Anticoagulants.

**Table 2:** Sex-specific incidence of cardiovascular comorbidities and incidence rate ratios.

	Men		Women		Incidence Rate Ratios Men/Women	
Incidence/1000 people per year (CI95%)	High AF-Risk (Q4th)	New AF	High AF-Risk (Q4th)	New AF	OR Q4th/Q4th (CI95%)	OR AF/AF (CI95%)
N	3151	863	4249	851		
AF Incidence/1000 people per year (CI95%)		9.33 (8.72–9.98)	-	7.24 (6.77–7.75)		1.28 (1.17–1.41) *p* < 0.001
Stroke/Transient ischemic attack Incidence/1000 people per year (CI95%)	310 4.27 (3.81–4.77)	133 6.7 (5.60–7.93)	352 3.60 (3.23–3.99)	125 6.37 (5.31–7.6)	1.18 (1.01–1.38) *p* < 0.030	1.04 (0.82–1.33) *p* = 0.7444
Heart Failure Incidence/1000 people per year (CI95%)	708 9.75 (9.05–10.50)	476 23.94 (21.84–26.2)	882 8.40 (7.83–8.99)	442 22.54 (20.5–24.74)	1.08 (0.98–1.2) *p* = 0.1224	1.06 (0.93–1.20) *p* = 0.3778
Ischemic Heart Disease Incidence/1000 people per year (CI95%)	816 11.24 (10.48–12.04)	235 11.82 (10.36–13.43)	536 5.48 (5.02–5.96)	141 7.2 (6.05–8.48)	2.05 (1.84–2.28) *p* < 0.001	1.64 (1.33–2.02) *p* < 0.001
Peripheral Arteriopathy Incidence/1000 people per year (CI95%)	753 10.37 (9.65–11.14)	216 10.87 (9.46–12.41)	470 4.80 (4.38–5.26)	122 6.22 (5.17–7.43)	2.16 (1.92–2.42) *p* < 0.001	1.74 (1.4–2.18) *p* < 0.001
Cognitive Impairment Incidence/1000 people per year (CI95%)	440 6.06 (5.51–6.66)	136 6.84 (5.74–8.09)	904 9.24 (8.64–9.86)	154 7.85 (6.66–9.20)	0.65 (0.58–0.73) *p* < 0.001	0.87 (0.7–1.1) *p* = 0.2650
Chronic Kidney Disease Incidence/1000 people per year (CI95%)	1042 14.36 (13.50–15.26)	359 18.06 (16.24–20.03)	1406 14.37 (13.62–15.14)	553 28.20 (25.9–30.65)	0.99 (0.90–1.08) *p* = 0.9965	0.64 (0.56–0.73) *p* < 0.001
Death all-causes Incidence/1000 people per year (CI95%)	1832 25.24 (24.10–26.46)	585 29.43 (27.09–31.91)	2865 29.27 (28.21–30.37)	563 28.71 (26.4–31.18)	0.86 (0.81–0.91) *p* < 0.001	1.02 (0.91–1.15) *p* = 0.6979

**Table 3:** Clinical outcomes per 1000 high-risk individuals (lifetime horizon).

Outcome	Usual Care	MATHIAS Strategy	Absolute Difference	Relative Reduction
**Overall population**				
MACE ^1^ events	190	125	–65	–34%
Ischemic strokes	78	52	–26	–33%
Severe post-stroke disability	36	26	–10	–28%
Cardiovascular deaths	42	30	–12	–29%
QALYs ^2^ (increment vs. usual care)	—	+74	—	—
**Women**				
MACE ^1^ events	205	132	−73	−36%
Ischemic strokes	90	58	−32	−36%
Severe post-stroke disability	44	31	−13	−30%
Cardiovascular deaths	40	29	−11	−28%
QALYs ^2^ (increment vs. usual care)		+78		
**Men**				
MACE ^1^ events	176	120	−56	−32%
Ischemic strokes	66	47	−19	−29%
Severe post-stroke disability	28	21	−7	−25%
Cardiovascular deaths	45	31	−14	−31%
QALYs ^2^ (increment vs. usual care)		+69		

^1.^ MACE, major adverse cardiovascular events; ^2^ QALYs, quality-adjusted life-years.

**Table 4:** Direct medical costs per 1000 high-risk individuals (€).

Cost Component	Usual Care	MATHIAS Strategy	Difference
**All**			
Screening and diagnostic costs	48,000	115,000	+67,000
Anticoagulation therapy	280,000	410,000	+130,000
Stroke acute care	1,755,000	1,045,000	–710,000
Long-term disability care	2,025,000	1,460,000	–565,000
Hospitalizations (non-stroke MACE)	730,000	530,000	–200,000
Total direct costs	4,838,000	3,560,000	–1,278,000
**Women**			
Total direct medical costs	5,020,000	3,620,000	−1,400,000
**Men**			
Total direct medical costs	4,610,000	3,610,000	−1,000,000

**Table 5:** Societal costs and productivity losses per 1000 individuals (€).

Component	Usual Care	MATHIAS Strategy	Difference
Informal caregiving	890,000	545,000	–345,000
Long-term social care	1,240,000	780,000	–460,000
Productivity losses (<75 years)	210,000	140,000	–70,000
Nursing-home admissions	650,000	390,000	–260,000
**Total societal costs**	2,990,000	1,855,000	–1,135,000
**Women**			
Total societal costs	3,150,000	1,750,000	−1,400,000
**Men**			
Total societal costs	2,780,000	1,780,000	−1,000,000

**Table 6:** Incremental cost effectiveness results (base case).

Outcome	Overall Population	Women	Men
Incremental costs (healthcare system)	–€1,278,000	−1,400,000	−1,000,000
Incremental QALYs ^1^	+74	+78	+69
ICER ^2^	Dominant	Dominant	Dominant
Probability cost-effective at €30,000/QALY	0.88	0.90	0.85
Probability cost-saving	0.73	0.76	0.69

^1^ QALYs, quality-adjusted life-years; ^2^ ICER, incremental cost-effectiveness ratio.

## Data Availability

The data that support the findings of this study are available from Institut Catala de la Salut, but restrictions apply to availability of these data, which were used under license for the current study, and so are not publicly available. Datasets generated and analyzed during the current study are available from the corresponding author on reasonable request and with permission of the Institut Catala de la Salut (sensitive data).

## Supplementary Materials

The following supporting information can be downloaded at: https://www.mdpi.com/article/10.3390/jcm15082861/s1, Figure S1: Terres Ebre Map; Table S1: Cohort ≥ 65 year-old Terres Ebre.

## Abbreviations

The following abbreviations are used in this manuscript:

ACC/AHA/ACCP/HRS guideline	American College of Cardiology, the American Heart Association, the American College of Clinical Pharmacy, and the Heart Rhythm Society.
AF	atrial Fibrillation.
AGAUR	Agency for Management of University and Research Grants.
AI	Artificial Intelligence.
AUC	Area Under the Curve.
CEIm IDIAPJGol	Jordi Gol Primary Care Research Institute Ethics Committee.
CHA_2_DS_2_-VA score	Congestive heart failure; Hypertension; Aged ≥ 75 years; Diabetes mellitus; Previous Stroke; Vascular disease; Aged 65–74 years.
CHEERS	Consolidated Health Economic Evaluation Reporting Standards.
HER	Electronic Health Records.
HCC3/CMBD	Catalonia Shared Medical Record/ Minimum Basic Data Set.
MACE	Major Adverse Cardiovascular Event.
MATHIAS	throMboembolic risk Associated To High atrIal fibrillation riSk.
PIP	Population Impact Plot.
QALYs	Quality-Adjusted Life-Years.
SAPiC	Primary and Community Health Care Service.
SEEs	extracranial systemic embolic events.

## References

[1] Luengo-Fernandez R., Violato M., Candio P., Leal J. (2020). Economic burden of stroke across Europe: A population-based cost analysis. Eur. Stroke J..

[2] Lucas-Noll J., Clua-Espuny J.L., Lleixà-Fortuño M., Gavaldà-Espelta E., Queralt-Tomas L., Panisello-Tafalla A., Carles-Lavila M. (2023). The costs associated with stroke care continuum: A systematic review. Health Econ. Rev..

[3] Ding L., Tang M. (2025). Global, regional and national burden of atrial fibrillation and flutter attributable to metabolic risks from 1990 to 2021: Analysis of data from the global burden of disease study, 2021. Europace.

[4] Siddiqi H.K., Vinayagamoorthy M., Gencer B., Ng C., Pester J., Cook N.R., Lee I.-M., Buring J., Manson J.E., Albert C.M. (2022). Sex Differences in Atrial Fibrillation Risk: The VITAL Rhythm Study. JAMA Cardiol..

[5] Proietti M., Romiti G.F., Vitolo M., Borgi M., Di Rocco A., Farcomeni A., Miyazawa K., Healey J.S., Lane D.A., Boriani G. (2022). Epidemiology of subclinical atrial fibrillation in patients with cardiac implantable electronic devices: A systematic review and meta-regression. Eur. J. Intern. Med..

[6] Clua-Espuny J.L., Hernández-Pinilla A., Gentille-Lorente D., Muria-Subirats E., Forcadell-Arenas T., de Diego-Cabanes C., Ribas-Seguí D., Diaz-Vilarasau A., Molins-Rojas C., Palleja-Millan M. (2025). Evidence Gaps and Lessons in the Early Detection of Atrial Fibrillation: A Prospective Study in a Primary Care Setting (PREFATE Study). Biomedicines.

[7] Ruiz-Cantero M.T., Vives-Cases C., Artazcoz L., Delgado A., Calvente M.d.M.G., Miqueo C., Montero I., Ortiz R., Ronda E., Ruiz I. (2007). A framework to analyse gender bias in epidemiological research. J. Epidemiol. Community Health.

[8] Vogel B., Acevedo M., Appelman Y., Bairey Merz C.N., Chieffo A., Figtree G.A., Guerrero M., Kunadian V., Lam C.S.P., Maas A.H.E.M. (2021). The Lancet Women and Cardiovascular Disease Commission: Reducing the Global Burden by 2030. Lancet.

[9] Al Hamid A., Beckett R., Wilson M., Jalal Z., Cheema E., Obe D.A.-J., Coombs T., Ralebitso-Senior K., Assi S. (2024). Gender Bias in Diagnosis, Prevention, and Treatment of Cardiovascular Diseases: A Systematic Review. Cureus.

[10] van Gelder I.C., Rienstra M., Bunting K.V., Casado-Arroyo R., Caso V., Crijns H.J.G.M., De Potter T.J.R., Dwight J., Guasti L., ESC Scientific Document Group (2024). 2024 ESC Guidelines for the management of atrial fibrillation developed in collaboration with the European Association for Cardio-Thoracic Surgery (EACTS). Eur. Heart J..

[11] Clua-Espuny J.L., Panisello-Tafalla A., Lucas-Noll J., Muria-Subirats E., Forcadell-Arenas T., Carrera-Ortiz J.M., Molto-Balado P., Clua-Queralt J., Fusté-Anguera I., Reverte-Vilarroya S. (2025). Stroke Risk Stratification in Incident Atrial Fibrillation: A Sex-Specific Evaluation of CHA2DS2-VA and CHA2DS2-VASc. J. Cardiovasc. Dev. Dis..

[12] Tanaka Y., Shah N.S., Passman R., Greenland P., Lloyd-Jones D.M., Khan S.S. (2021). Trends in Cardiovascular Mortality Related to Atrial Fibrillation in the United States, 2011 to 2018. J. Am. Heart Assoc..

[13] Ko D., Rahman F., Schnabel R.B., Yin X., Benjamin E.J., Christophersen I.E. (2016). Atrial fibrillation in women: Epidemiology, pathophysiology, presentation, and prognosis. Nat. Rev. Cardiol..

[14] Joglar J.A., Chung M.K., Armbruster A.L., Benjamin E.J., Chyou J.Y., Cronin E.M., Deswal A., Eckhardt L.L., Goldberger Z.D., Gopinathannair R. (2023). 2023 ACC/AHA/ACCP/HRS Guideline for the Diagnosis and Management of Atrial Fibrillation: A Report of the American College of Cardiology/American Heart Association Joint Committee on Clinical Practice Guidelines. Circulation.

[15] Moltó-Balado P., Reverté-Villarroya S., Alonso-Barberán V., Monclús-Arasa C., Balado-Albiol M.T., Clua-Queralt J., Clua-Espuny J.L. (2024). Machine Learning Approaches to Predict Major Adverse Cardiovascular Events in Atrial Fibrillation. Technologies.

[16] Moltó-Balado P., Clua-Espuny J.-L., Reverté-Villarroya S., Alonso-Barberán V., Balado-Albiol M.T., Simeó-Monzó A., Canela-Royo J., del Barrio-González A. (2025). Prediction of Major Adverse Cardiovascular Events in Atrial Fibrillation: A Comparison Between Machine Learning Techniques and CHA2DS2-VA Score. Inventions.

[17] Moltó-Balado P., Reverté-Villarroya S., Monclús-Arasa C., Balado-Albiol M.T., Baset-Martínez S., Carot-Domenech J., Clua-Espuny J.L. (2023). Heart Failure and Major Adverse Cardiovascular Events in Atrial Fibrillation Patients: A Retrospective Primary Care Cohort Study. Biomedicines.

[18] Clúa-Espuny J.L., Panisello-Tafalla A., Hernández-Pinilla A., Clua-Queralt J., Múria-Subirats E., Lucas-Noll J., Moltó-Balado P., Forcadell-Arenas T., Reverté-Villarroya S. (2025). Sex Differences in Individuals at High Risk of Atrial Fibrillation: A Primary Care Community Cohort Study, 2015-2024. Biomedicines.

[19] Generalitat de Catalunya (2008). Projeccions de Població Principals Resultats 2013–2051. https://www.idescat.cat/serveis/biblioteca/docs/cat/pp2021-2041pr.pdf.

[20] Idescat Anuario Estadístico de Cataluña. Renda Familiar Disponible Bruta. Índex. Comarques i Aran, i Àmbits. http://www.idescat.cat/pub/?id=aec&n=941.

[21] Palà E., Bustamante A., Clúa-Espuny J.L., Acosta J., González-Loyola F., Dos Santos S., Ribas-Segui D., Ballesta-Ors J., Penalba A., Giralt M. (2022). Blood-biomarkers and devices for atrial fibrillation screening: Lessons learned from the AFRICAT (Atrial Fibrillation Research In CATalonia) study. PLoS ONE.

[22] Abellana R., Gonzalez-Loyola F., Verdu-Rotellar J.M., Bustamante A., Palà E., Clua-Espuny J.L., Montaner J., Pedrote A., del Val-Garcia J.L., Segui D.R. (2021). Predictive model for atrial fibrillation in hypertensive diabetic patients. Eur. J. Clin. Investig..

[23] Hernández-Pinilla A., Clua-Espuny J.L., Satué-Gracia E.M., Pallejà-Millán M., Martín-Luján F.M. (2024). Protocol for a multicentre and prospective follow-up cohort study of early detection of atrial fibrillation, silent stroke and cognitive impairment in high-risk primary care patients: The PREFA-TE study. BMJ Open.

[24] Queralt-Tomas L., Clua-Espuny J.L., Fernández-Saez J., Lleixà-Fortuño M.M., Albiol-Zaragoza I., Gil-Guillen V., Carratala-Munuera C. (2019). Risk of Dependency: A Challenge for Health and Social Care Planning-Observational Stroke Cohort. Value Health.

[25] Khurshid S., Friedman S.F., Al-Alusi M.A., Kany S., Sommers T., Anderson C.D., Ho J.E., McManus D.D., Borowsky L.H., Ashburner J.M. (2025). Artificial intelligence-enabled analysis of handheld single-lead electrocardiograms to predict incident atrial fibrillation: An analysis of the VITAL-AF randomized trial. npj Digit. Med..

[26] Pedroso A.F., Khera R. (2025). Leveraging AI-enhanced digital health with consumer devices for scalable cardiovascular screening, prediction, and monitoring. npj Cardiovasc. Health.

[27] Eklund M., Bernfort L., Appelberg K., Engler D., Schnabel R.B., Martinez C., Wallenhorst C., Boriani G., Buckley C.M., Diederichsen S.Z. (2024). The budget impact of implementing atrial fibrillation-screening in European countries. Eur. Heart J. Suppl..

[28] Abolghasem Gorji H., Khosravi M., Mahmoodi R., Hasoumi M., Souresrafil A., Alipour V., Rezapour A., Hajahmadi M., Azari S. (2023). Cost-Effectiveness of Atrial Fibrillation Screening Strategies: A Systematic Review. Iran. J. Public Health.

[29] Lu W.D., Chen J.Y. (2021). Atrial high-rate episodes and risk of major adverse cardiovascular events in patients with dual chamber permanent pacemakers: A retrospective study. Sci. Rep..

[30] Gupta M., Parra C.M., Dennehy D. (2022). Questioning Racial and Gender Bias in AI-based Recommendations: Do Espoused National Cultural Values Matter?. Inf. Syst. Front..

[31] Khan M., Ingre M., Carlstedt F., Eriksson A., Skröder S., Tenn J.S., Rosenqvist M., Svennberg E. (2024). Increasing the reach: Optimizing screening for atrial fibrillation-the STROKESTOP III study. Europace.

[32] Adedinsewo D.A., Pollak A.W., Phillips S.D., Smith T.L., Svatikova A., Hayes S.N., Mulvagh S.L., Norris C., Roger V.L., Noseworthy P.A. (2022). Cardiovascular Disease Screening in Women: Leveraging Artificial Intelligence and Digital Tools. Circ. Res..

[33] Nakamizo T., Misumi M., Takahashi T., Kurisu S., Matsumoto M., Tsujino A. (2023). Female “Paradox” in Atrial Fibrillation—Role of Left Truncation Due to Competing Risks. Life.

[34] Moltó-Balado P., Clua-Espuny J.L., Tarongi-Vidal C., Barrios-Carmona P., Alonso-Barberán V., Balado-Albiol M.T., Simeó-Monzó A., Canela-Royo J., Del Barrio-González A. (2025). Risk of Chronic Kidney Disease and Implications in Patients with Atrial Fibrillation for the Development of Major Adverse Cardiovascular Events with Machine Learning. Med. Sci..

[35] Hill N.R., Sandler B., Mokgokong R., Lister S., Ward T., Boyce R., Farooqui U., Gordon J. (2020). Cost-effectiveness of targeted screening for the identification of patients with atrial fibrillation: Evaluation of a machine learning risk prediction algorithm. J. Med. Econ..

[36] Engler D., Heidbuchel H., Schnabel R.B. (2021). For the AFFECT-EU Investigators, Digital, risk-based screening for atrial fibrillation in the European community—The AFFECT-EU project funded by the European Union. Eur. Heart J..

