# Supplementary material for: Sex-Specific Health and Economic Benefits in Older Women at Risk of Atrial Fibrillation: A Proof-of-Concept Evaluation of an AI-Enabled Strategy for Early Thromboembolic Risk Detection
Source: J Clin Med. 2026 Apr 9;15(8):2861. doi: 10.3390/jcm15082861 (PMC13115981; doi:10.3390/jcm15082861)
Supplement: Supplementary file 1 [file jcm-15-02861-s001.zip › jcm-4216864-supplementary.pdf]

**Supplementary Table S1. Baseline characteristics of the study population. (n = 40,079)**

| Variables | MEN | (%) | WOMEN | (%) | p | ALL (%) |
|---|---|---|---|---|---|---|
| All (n %) | 19,531 | 48.7% | 20,548 | 51.3% | - | 40,079 |
| New AF | 1,928 | 9.9% | 1,442 | 7.0% | < 0.001 | 3,370 (8.4%) |
| Age average | 77.28±6.56 | | 77.6±6.63 | | < 0.001 | 77.4±6.60 |
| $CHA_2DS_2$-VA | 2.8±1.1 | | 2.6±1.1 | | < 0.001 | 2.68±1.1 |
| Heart failure | 1,790 | 9.2% | 1,558 | 7.6.·% | < 0.001 | 3,348 (8.4%) |
| Hypertension arterial | 11,526 | 59.0% | 12,218 | 59.5% | 0.360 | 23,744 (59.2%) |
| Age 65 to 74 years | 7,748 | 39.6% | 7,765 | 37.8% | < 0.001 | 15,513 (38.7%) |
| Age ≥75 years | 11,783 | 60.3% | 12,783 | 62.6% | < 0.001 | 24,566 (61.3%) |
| Diabetes mellitus | 5,763 | 29.5% | 4,498 | 21.9% | < 0.001 | 10,261 (25.6%) |
| Stroke/TIA/Systemic embolism | 843 | 4.7% | 724 | 3.5% | < 0.001 | 1567 (3.9%) |
| Peripheral vascular disease | 1,983 | 10.2% | 798 | 3.9% | < 0.001 | 2,781 (6.9%) |
| Ischemic heart disease | 2,073 | 10.6% | 917 | 4.5% | < 0.001 | 2,990 (7.5%) |
| BMI[1] ($kg/m^2$) | 28.1±4.5 | | 28.4±5.7 | | < 0.001 | 28.3±5.2 |
| Charlson index | 1.5±1.4 | | 1.2±1.2 | | < 0.001 | 1.38±1.9 |
| Dementia/cognitive impairment | 1,452 | 7.4% | 2,225 | 10.8% | < 0.001 | 3,677 (9.2%) |
| Pfeiffer score | 2.72±3.20 | | 3.63±3.35 | | < 0.001 | 3.23±3.3 |
| Chronic Kidney Disease | 3,181 | 16.3% | 3,080 | 15.0% | < 0.001 | 6,261 (15.6%) |
| Glomerular filtration rate (ml/min/1.73 $m^2$) | 72.2±18.0 | | 73.4±17.6 | | < 0.001 | 72.9±17.7 |
| OSAHS[2] | 966 | 4.9% | 473 | 2.3% | < 0.001 | 1,439 (3.6%) |
| Dyslipidemia | 8,394 | 43.0% | 10,623 | 51.7% | < 0.001 | 19,017 (47.5%) |
| Statins | 6,006 | 30.8% | 6,326 | 30.8% | 0.940 | 12,332 (30.8%) |
| Antiplatelet therapy | 3,411 | 17.5% | 2,335 | 11.4% | < 0,001 | 5,746 (14.3%) |
| Anticoagulation | 1,977 | 10.1% | 1,468 | 7.1% | < 0.001 | 3,445 (8.6%) |
| Hospital visits | 0.36±1.3 | | 0.27±0.95 | | < 0.001 | 0.31±1,15 |
| Active medications | 5.17±4.3 | | 5.76±4.40 | | < 0.001 | 5.48±4.36 |
| Death all-causes | 14,492 | 74.2% | 17,173 | 83.6% | < 0.001 | 31,665 (79.0%) |

BMI[1] (kg/m2): Body Mass Index; OSAHS[2]: Obstructive Sleep Apnea-Hypopnea Syndrome

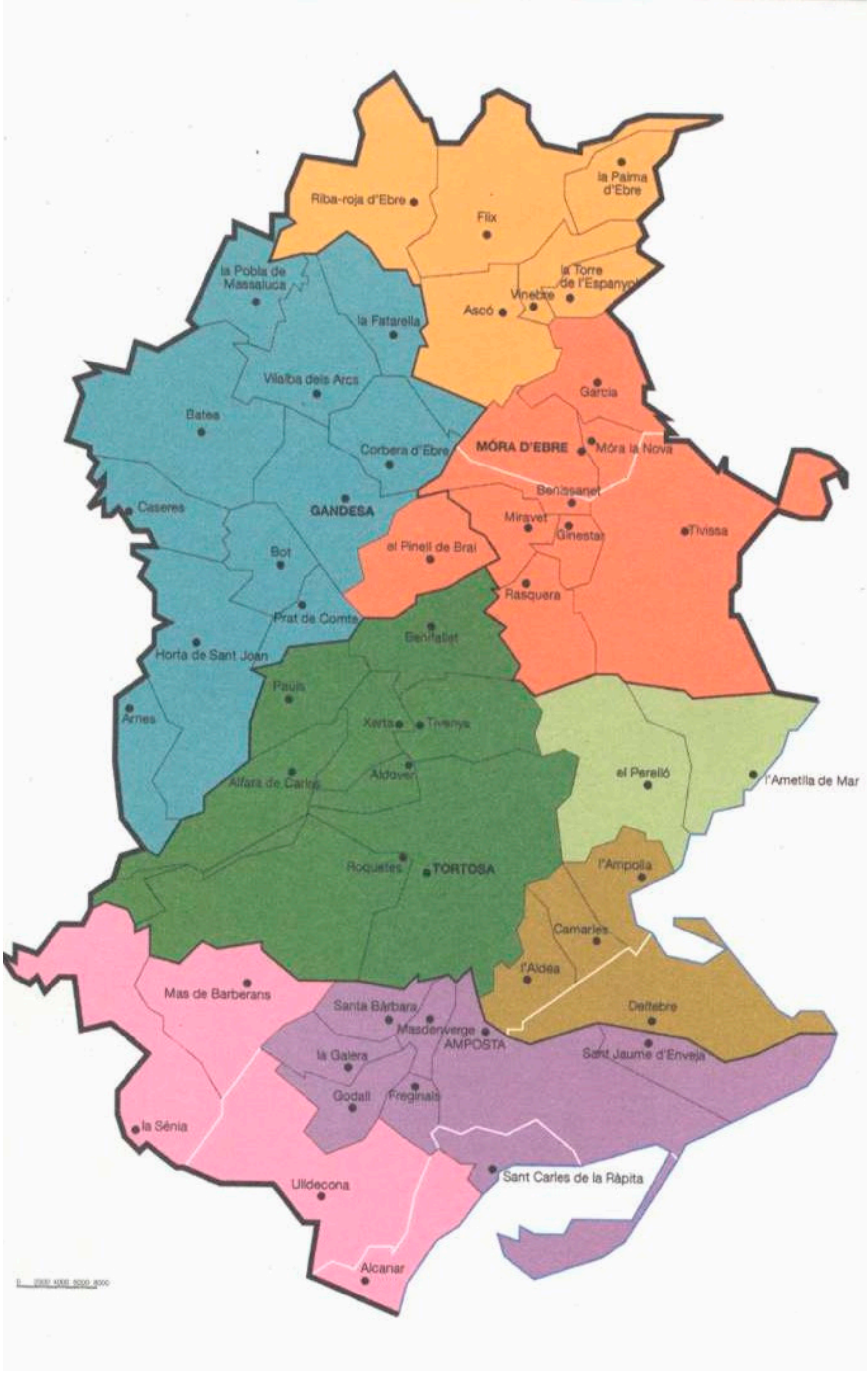


**Figure S1.** Terres Ebre Map.
